# Supplementary material for: Quantifying spontaneous infant movements using state-space models
Source: Sci Rep. 2024 Nov 19;14:28598. doi: 10.1038/s41598-024-80202-x (PMC11576873; doi:10.1038/s41598-024-80202-x)
Supplement: Supplementary file 1 — Supplementary Material 1 [file 41598_2024_80202_MOESM1_ESM.docx]

# SUPPLEMENTAL MATERIAL

# Quantifying spontaneous infant movements using state-space models


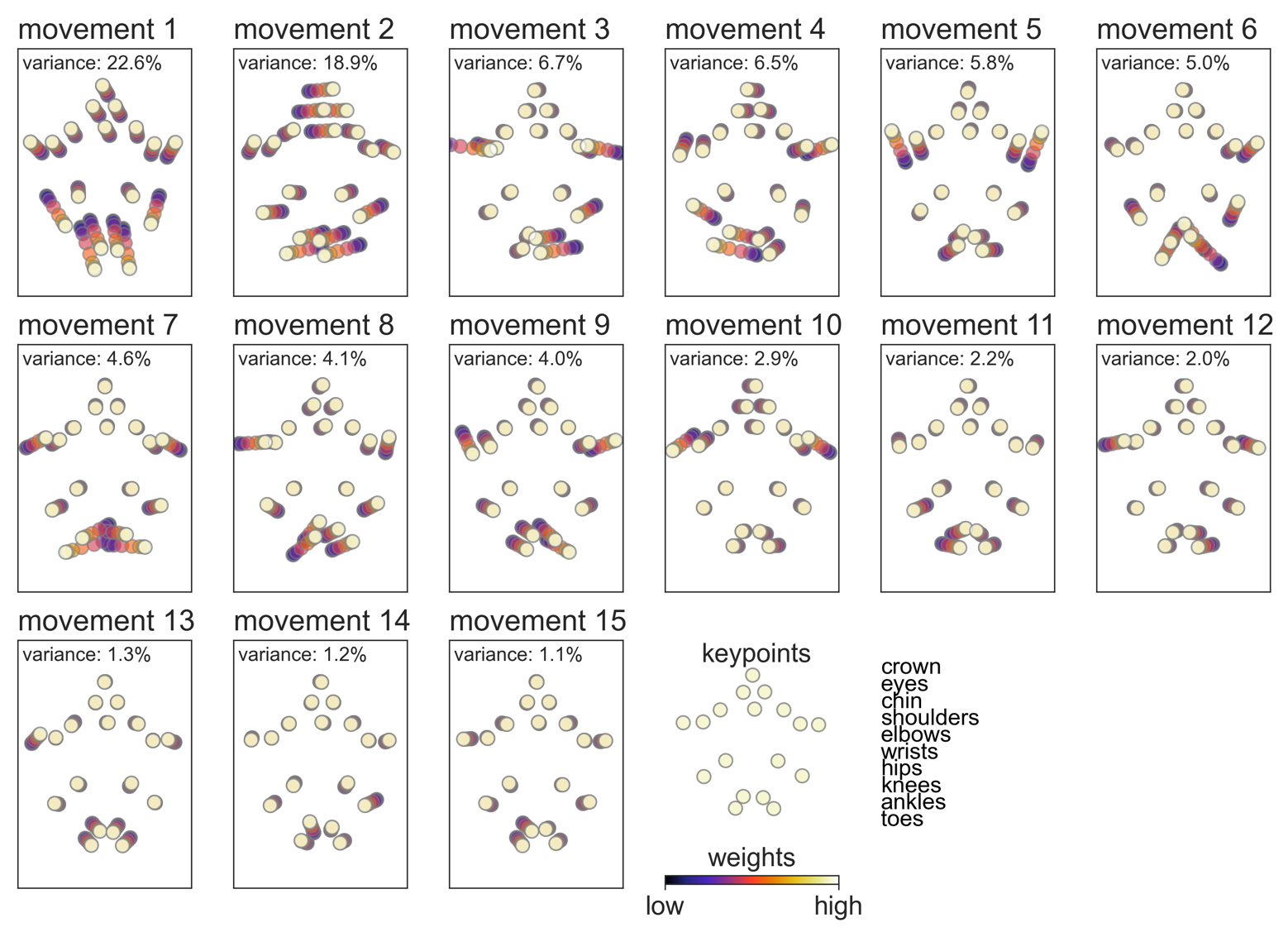


**Figure S1: Principal movements.** Each plot shows one principal movement (PM). The position of each keypoint at a given PM weight is shown in colour (colourbar, arbitrary scale).

**
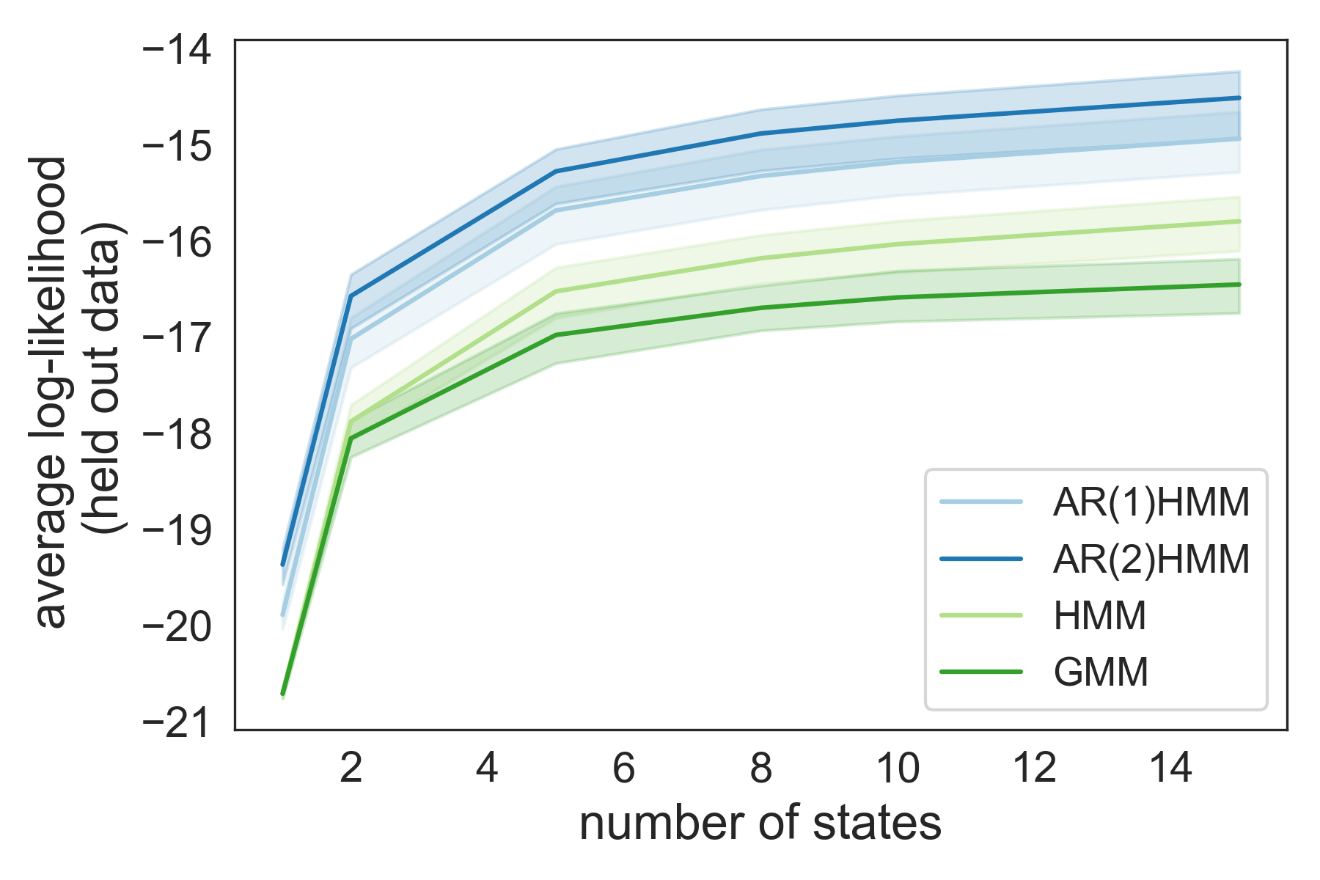
**

**Figure S2: Goodness-of-fit for HMM and GMM models.** Average log-likelihood on the test set across 5 cross-validation folds is shown with 95% confidence intervals (shaded) for each model type.

**
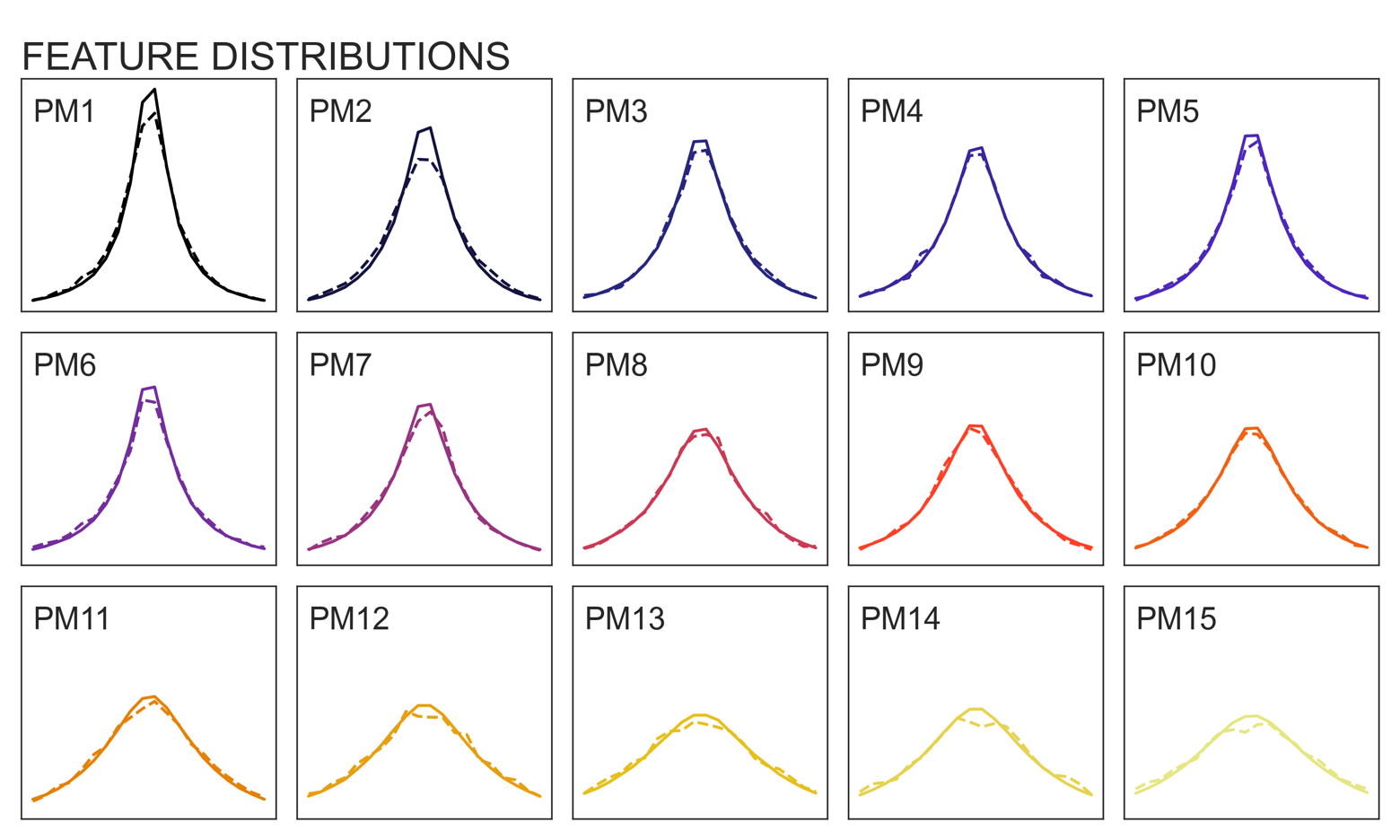
**

**Figure S3: Distribution of PM weights in empirical (solid) and synthetic (dashed) data.**


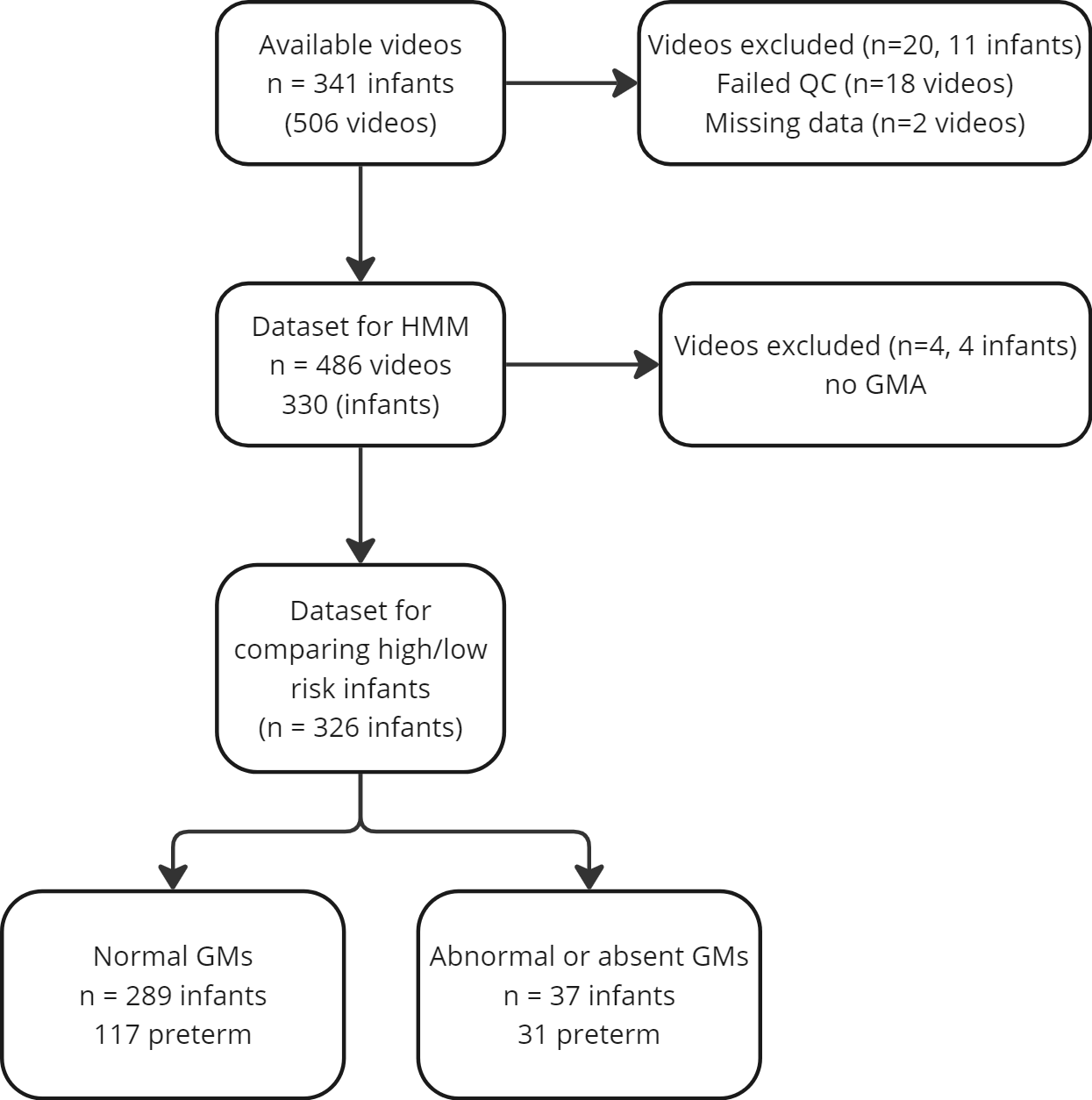


**Figure S4: Flow chart of participants and videos for analysis.** GM: general movements; GMA: General Movement Assessment; HMM: Hidden Markov Model; QC: quality control protocol, see methods for details.

| **Table S1: model fit averaged across cross-validation folds** | | | | |
| --- | --- | --- | --- | --- |
| **model** | **states** | **lag** | **log-likelihood** | **AIC** |
| GMM | 1 | - | -20.71 | 1490884 |
|  | 2 | - | -18.05 | 1300229 |
|  | 5 | - | -16.98 | 1223700 |
|  | 8 | - | -16.70 | 1204180 |
|  | 10 | - | -16.59 | 1196997 |
|  | 15 | - | -16.45 | 1188694 |
| HMM | 1 | - | -20.71 | 1491098 |
|  | 2 | - | -17.88 | 1288119 |
|  | 5 | - | -16.53 | 1192168 |
|  | 8 | - | -16.18 | 1168899 |
|  | 10 | - | -16.04 | 1159374 |
|  | 15 | - | -15.80 | 1144988 |
| AR(1)HMM | 1 | 1 | -19.89 | 1432452 |
|  | 2 | 1 | -17.02 | 1227143 |
|  | 5 | 1 | -15.68 | 1133670 |
|  | 8 | 1 | -15.33 | 1110840 |
|  | 10 | 1 | -15.18 | 1102360 |
|  | 15 | 1 | -14.94 | 1089789 |
| AR(2)HMM | 1 | 2 | -19.37 | 1395196 |
|  | 2 | 2 | -16.57 | 1195808 |
|  | 5 | 2 | -15.28 | 1106586 |
|  | 8 | 2 | -14.88 | 1082540 |
|  | 10 | 2 | -14.75 | 1075781 |
|  | 15 | 2 | -14.51 | 1065919 |

| **Table S2: Fixed effects model terms for age-related changes in state occupancy** | | | | | |
| --- | --- | --- | --- | --- | --- |
| **model terms** | **intercept** |  | **age** |  |  |
| **state** | **β** | **p** | **β** | **p** |  |
| 1 | 137.62 | 6.47×10^-6^ | 4.65 | 3.10×10^-2^ |  |
| 2 | 208.04 | 7.35×10^-8^ | 3.91 | 1.53×10^-1^ |  |
| 3 | 318.90 | 8.58×10^-14^ | -3.43 | 2.57×10^-1^ |  |
| 4 | 364.26 | 1.48×10^-17^ | -7.55 | 1.26×10^-2^ |  |
| 5 | 339.41 | 5.01×10^-30^ | -9.26 | 1.08×10^-5^ |  |
| 6 | 153.50 | 1.95×10^-3^ | 5.27 | 1.33×10^-1^ |  |
| 7 | 46.11 | 1.91×10^-2^ | 7.23 | 2.02×10^-7^ |  |
| 8 | 229.15 | 4.80×10^-13^ | -0.66 | 7.68×10^-1^ |  |

| **Table S3: Fixed effects model terms for associations with GMA outcome** | | | | | | | | |  | |  | |  | |  |
| --- | --- | --- | --- | --- | --- | --- | --- | --- | --- | --- | --- | --- | --- | --- | --- |
| **model terms** | **intercept** | | **age** | |  | | **birth status** | |  | | **GMA outcome** | | | |  |
| **state** | **β** | **p** | | **β** | | **p** | | **β** | | **p** | | **β** | | **p** | |
| 1 | 124.66 | 1.22×10^-4^ | | 4.58 | | 3.65×10^-2^ | | -31.85 | | 1.11×10^-4^ | | 3.00 | | 8.14×10^-1^ | |
| 2 | 212.34 | 2.21×10^-7^ | | 4.32 | | 1.22×10^-1^ | | 21.52 | | 2.86×10^-2^ | | -2.41 | | 8.74×10^-1^ | |
| 3 | 288.88 | 1.16×10^-10^ | | -4.89 | | 1.10×10^-1^ | | -35.81 | | 7.81×10^-4^ | | -34.00 | | 3.87×10^-2^ | |
| 4 | 395.04 | 1.52×10^-18^ | | -6.15 | | 4.49×10^-2^ | | 38.69 | | 2.99×10^-4^ | | 32.77 | | 4.72×10^-2^ | |
| 5 | 340.24 | 7.25×10^-27^ | | -8.72 | | 4.21×10^-5^ | | 26.82 | | 1.09×10^-3^ | | -7.83 | | 5.38×10^-1^ | |
| 6 | 150.43 | 4.38×10^-3^ | | 4.73 | | 1.82×10^-1^ | | 22.43 | | 1.04×10^-1^ | | -23.31 | | 2.76×10^-1^ | |
| 7 | 61.40 | 3.47×10^-3^ | | 7.33 | | 2.65×10^-7^ | | -7.80 | | 1.26×10^-1^ | | 23.72 | | 2.64×10^-3^ | |
| 8 | 222.96 | 2.60×10^-11^ | | -0.98 | | 6.68×10^-1^ | | -34.38 | | 1.65×10^-5^ | | 8.45 | | 4.93×10^-1^ | |

| **Table S4: Fixed effects model terms for associations with Bayley Motor scores** | | | | | |  |  |  |
| --- | --- | --- | --- | --- | --- | --- | --- | --- |
| **model terms** | **intercept** |  | **age** |  | **birth status** |  | **Motor** |  |
| **state** | **β** | **p** | **β** | **p** | **β** | **p** | **β** | **p** |
| 1 | 178.29 | 1.23×10^-5^ | 2.83 | 1.95×10^-1^ | -31.66 | 1.80×10^-4^ | -0.32 | 2.07×10^-1^ |
| 2 | 175.73 | 6.26×10^-4^ | 4.31 | 1.25×10^-1^ | 20.48 | 4.65×10^-2^ | 0.36 | 2.39×10^-1^ |
| 3 | 223.25 | 8.42×10^-5^ | -1.86 | 5.50×10^-1^ | -30.29 | 7.70×10^-3^ | 0.54 | 1.10×10^-1^ |
| 4 | 469.51 | 2.77×10^-16^ | -8.68 | 5.38×10^-3^ | 32.68 | 4.74×10^-3^ | -0.67 | 5.09×10^-2^ |
| 5 | 333.96 | 6.63×10^-16^ | -8.64 | 6.47×10^-5^ | 24.72 | 4.94×10^-3^ | 0.11 | 6.65×10^-1^ |
| 6 | 138.09 | 4.61×10^-2^ | 6.87 | 5.74×10^-2^ | 20.57 | 1.65×10^-1^ | 0.03 | 9.43×10^-1^ |
| 7 | 93.19 | 3.24×10^-4^ | 6.34 | 5.51×10^-6^ | -9.11 | 8.33×10^-2^ | -0.39 | 1.39×10^-2^ |
| 8 | 185.48 | 1.05×10^-5^ | -1.19 | 6.05×10^-1^ | -27.59 | 1.08×10^-3^ | 0.35 | 1.69×10^-1^ |

| **Table S5: Fixed effects model terms for associations with Bayley Cognitive scores** | | | | | | |  |  |
| --- | --- | --- | --- | --- | --- | --- | --- | --- |
| **model terms** | **intercept** |  | **age** |  | **birth status** |  | **Cognitive** |  |
| **state** | **β** | **p** | **β** | **p** | **β** | **p** | **β** | **p** |
| 1 | 206.30 | 7.61×10^-7^ | 2.74 | 2.09×10^-1^ | -33.80 | 4.61×10^-5^ | -0.59 | 2.88×10^-2^ |
| 2 | 178.04 | 7.25×10^-4^ | 4.27 | 1.28×10^-1^ | 19.61 | 5.38×10^-2^ | 0.35 | 2.97×10^-1^ |
| 3 | 197.08 | 6.94×10^-4^ | -1.78 | 5.66×10^-1^ | -28.84 | 9.73×10^-3^ | 0.80 | 2.85×10^-2^ |
| 4 | 521.72 | 3.82×10^-19^ | -8.80 | 4.64×10^-3^ | 28.92 | 1.04×10^-2^ | -1.19 | 1.24×10^-3^ |
| 5 | 355.22 | 6.41×10^-17^ | -8.72 | 5.53×10^-5^ | 22.45 | 9.79×10^-3^ | -0.09 | 7.55×10^-1^ |
| 6 | 75.98 | 2.85×10^-1^ | 7.08 | 4.97×10^-2^ | 26.59 | 6.87×10^-2^ | 0.63 | 1.87×10^-1^ |
| 7 | 71.19 | 7.69×10^-3^ | 6.45 | 4.20×10^-6^ | -6.29 | 2.29×10^-1^ | -0.18 | 2.85×10^-1^ |
| 8 | 191.68 | 8.97×10^-6^ | -1.24 | 5.89×10^-1^ | -28.81 | 5.54×10^-4^ | 0.29 | 2.81×10^-1^ |

| **Table S6: Fixed effects model terms for associations with Bayley Language scores** | | | | | | |  |  |
| --- | --- | --- | --- | --- | --- | --- | --- | --- |
| **model terms** | **intercept** |  | **age** |  | **birth status** |  | **Language** |  |
| **state** | **β** | **p** | **β** | **p** | **β** | **p** | **β** | **p** |
| 1 | 222.51 | 1.29×10^-8^ | 2.20 | 3.27×10^-1^ | -36.29 | 4.30×10^-5^ | -0.67 | 3.02×10^-3^ |
| 2 | 183.76 | 2.58×10^-4^ | 3.96 | 1.76×10^-1^ | 22.22 | 4.46×10^-2^ | 0.36 | 2.08×10^-1^ |
| 3 | 202.84 | 2.58×10^-4^ | -1.55 | 6.30×10^-1^ | -25.22 | 3.86×10^-2^ | 0.72 | 1.96×10^-2^ |
| 4 | 502.20 | 4.08×10^-20^ | -9.52 | 2.83×10^-3^ | 35.89 | 2.74×10^-3^ | -0.85 | 5.10×10^-3^ |
| 5 | 342.36 | 2.66×10^-17^ | -9.28 | 4.78×10^-5^ | 31.04 | 9.68×10^-4^ | 0.19 | 4.40×10^-1^ |
| 6 | 70.37 | 2.80×10^-1^ | 8.07 | 2.85×10^-2^ | 13.10 | 3.87×10^-1^ | 0.41 | 2.87×10^-1^ |
| 7 | 81.39 | 9.84×10^-4^ | 6.66 | 2.59×10^-6^ | -11.93 | 3.08×10^-2^ | -0.35 | 1.30×10^-2^ |
| 8 | 193.93 | 2.87×10^-6^ | -0.66 | 7.84×10^-1^ | -28.89 | 1.72×10^-3^ | 0.21 | 3.73×10^-1^ |
